# The role of PB1-F2 in adaptation of high pathogenicity avian influenza virus H7N7 in chickens

**DOI:** 10.1186/s13567-023-01257-8

**Published:** 2024-01-03

**Authors:** Luise Hohensee, David Scheibner, Alexander Schäfer, Holly Shelton, Thomas C. Mettenleiter, Angele Breithaupt, Anca Dorhoi, Elsayed M. Abdelwhab, Ulrike Blohm

**Affiliations:** 1 Institute of Immunology, Friedrich-Loeffler-Institut, Federal Research Institute for Animal Health, Südufer 10, 17493 Greifswald, Insel Riems Germany; 2 Institute of Molecular Virology and Cell Biology, Friedrich-Loeffler-Institut, Federal Research Institute for Animal Health, Südufer 10, 17493 Greifswald, Insel Riems Germany; 3 The Pirbright Institute, Pirbright, Ash Road, Surrey, GU24 0NF UK; 4 Friedrich-Loeffler-Institut, Federal Research Institute for Animal Health, Südufer 10, 17493 Greifswald, Insel Riems Germany; 5 Department of Experimental Animal Facilities and Biorisk Management, Friedrich-Loeffler-Institut, Federal Research Institute for Animal Health, Südufer 10, 17493 Greifswald, Insel Riems Germany; 6 Present Address: Infection Pathogenesis, TUM School of Life Sciences, Technische Universität München, 85354 Freising, Germany

**Keywords:** PB1-F2, H7N7, chicken, apoptosis, cellular immunity, virulence, survival, tissue tropism, replication, polymerase

## Abstract

Avian influenza viruses (AIV) of the H7N7 subtype are enzootic in the wild bird reservoir in Europe, cause infections in poultry, and have sporadically infected humans. The non-structural protein PB1-F2 is encoded in a second open frame in the polymerase segment PB1 and its sequence varies with the host of origin. While mammalian isolates predominantly carry truncated forms, avian isolates typically express full-length PB1-F2. PB1-F2 is a virulence factor of influenza viruses in mammals. It modulates the host immune response, causing immunopathology and increases pro-inflammatory responses. The role of full-length PB1-F2 in IAV pathogenesis as well as its impact on virus adaptation and virulence in poultry remains enigmatic. Here, we characterised recombinant high pathogenicity AIV (HPAIV) H7N7 expressing or lacking PB1-F2 in vitro and in vivo in chickens. In vitro, full-length PB1-F2 modulated viability of infected chicken fibroblasts by limiting apoptosis. In chickens, PB1-F2 promoted gastrointestinal tropism, as demonstrated by enhanced viral replication in the gut and increased cloacal shedding. PB1-F2’s effects on cellular immunity however were marginal. Overall, chickens infected with full-length PB1-F2 virus survived for shorter periods, indicating that PB1-F2 is also a virulence factor in bird-adapted viruses.

**Supplementary Information:**

The online version contains supplementary material available at 10.1186/s13567-023-01257-8.

## Introduction

Influenza A viruses (IAV) are RNA viruses belonging to the *Orthomyxoviridae* family, which also comprises the genera Influenza B, C, and D [1]. While most Influenza viruses have a limited host range, IAV infect a wide spectrum of host species, including birds and mammals. Avian Influenza viruses (AIV) are prevalent in wild bird populations [2]. Aquatic birds are the natural reservoir and spread the virus globally along their migration routes [3]. AIV are divided according to variations of the surface glycoproteins, hemagglutinin (HA or H) and neuraminidase (NA or N), into 16 HA and 9 NA subtypes. While normally AIV exhibit low pathogenicity (LP) in poultry, LP H5/H7 subtypes can transform to high pathogenicity (HP) AIV and cause high and rapid mortality [4]. With the exception of the current panzootic H5Nx Goose/Guangdong lineage, HP H7 outbreaks are more prevalent than HP H5 outbreaks [5]. H7N9 is endemic in China since 2013 [6], and H7N3 is endemic in Mexico since 2012 [7, 8], in addition to several H7N7 outbreaks in Europe and Asia. H7 viruses show a broad species range and infect, besides wild and domestic birds, also harbour seals, horses, pigs, and humans [9]. The majority of human cases with confirmed IAV H7 infection present as mild or asymptomatic, yet several epidemics have demonstrated that such viruses are a non-negligible zoonotic threat, especially for those working closely with poultry [10–12].

AIV possess a segmented genome encoding several structural and non-structural proteins, including PB1-F2, which is expressed from an alternative open reading frame (ORF) of the polymerase segment PB1. Interestingly, PB1-F2 is only present in IAV, while Influenza B viruses do not express PB1-F2 [13]. It is a non-structural protein that is only detected in infected cells and has not yet been found in purified virions [14]. PB1-F2 is mostly 90 amino acids (aa) long, however, it can vary in length. Mutations result in the expression of truncated forms by either deleting the start codon [13] or by introducing premature stop codons at various position of the PB1-F2 ORF (e.g. positions 12, 25, 26, 38 and 58) [15]. Likewise, extended forms (up to 106 aa) have been reported [16]. The expression pattern of PB1-F2 is host-dependent. AIV typically carry the full-length protein [17], whereas PB1-F2 is under a strong negative selection pressure in mammal-adapted IAV [18]. The biological functions attributed to PB1-F2 expression are strain- and cell-specific. In mammalian hosts, full-length PB1-F2 has been associated with induction of apoptosis [13], increased polymerase activity [19], and enhanced virulence in mice [20], that causes increased vulnerability to bacterial superinfections [21]. It has been shown to interfere with interferon type I pathways [22, 23] and can stimulate immunopathology [24]. Despite the high prevalence of full-length PB1-F2 in AIV, its function in avian hosts is not fully understood. Some studies have shown that full-length PB1-F2 reduced virulence (H9N2 A/chicken/Pakistan/UDL01/08 [25], H5N1 A/duck/Niger/2090/2006 [26]), while others reported increased virulence (H5N1 A/Viet Nam/1203/2004 [27]) or no impact on disease outcome [28]. These inconsistent results suggest that the role of PB1-F2 in maintaining virus fitness depends heavily on virus subtype and experimental conditions. Most of these studies investigated H5 and H9 AIV, while data on the role of PB1-F2 in the fitness of H7 viruses is scarce. Considering the high prevalence of full-length PB1-F2 in potentially zoonotic AIV, there is an imminent risk of introduction into a vulnerable human population. A better understanding of the roles of this protein in the natural host could contribute to prevention of avian-to-human spill-over, particularly considering its potential role in viral shedding [26].

Here, we utilize HPAIV H7N7 expressing full-length (designated hereafter as wt) or lacking (designated hereafter as ΔF2) PB1-F2 to investigate the role of this non-structural protein in adaptation in vitro and in vivo. We hypothesize that full-length PB1-F2 may be evolutionary beneficial for IAV in avian hosts.

## Materials and methods

### Cells

MDCK-II (Madin-Darby canine kidney cells), HEK293T (human embryo kidney cells) and DF-1 (chicken fibroblasts) were sourced from the Collection of Cell Lines in Veterinary Medicine (CCLV) at the FLI. Chicken embryo kidney (CEK) cells were prepared as previously done [29]. Briefly, after incubating for 18–19 days at 37 °C chicken embryos were humanly killed, kidneys were harvested, cut, trypsinized and strained, producing a cell suspension.

The cells were cultivated using media also provided by CCLV: Confluent cells were treated with trypsin; detached cells were then resuspended in their respective media. A combination of MEM (Hank’s salts), MEM (Earle’s salts) and NEA were used for MDCK-II. DF-1 and HEK293T were cultivated in Ham’s F12 and IMDM (1:1). For all cells 10% fetal bovine serum (FBS) (PAN biotech) and 1% penicillin/streptomycin (100×) were added to the media. The cells were incubated at 37 °C and 5% CO_2_. CEK cells were seeded using MEM (Earle’s salts), 10% FBS, 1% P/S and 0.1% Amphotericin B in plates, that were previously treated with gelatine.

### Viruses

All experiments were conducted with high pathogenicity (HP) H7N7 A/chicken/Germany/AR1385/2015. The virus was previously isolated and characterized by Dietze et al. [30]. The recombinant HP H7N7 viruses expressing full-length PB1-F2 (wt) as well as the respective plasmids were previously described [31]. The knockout virus (ΔF2) was created by introducing a point mutation (C138A) to PB1 using the QuikChange Site-directed mutagenesis kit (Invitrogen). This mutation was silent in the PB1 reading frame, but changed Serine to a stop codon and truncated PB1-F2 at 12 amino acids (aa) length as previously described by James et al. [25].

All recombinant viruses were rescued and propagated as previously described [32, 33]. Briefly, embryonated chicken eggs were inoculated with supernatant from a transfected co-culture of HEK293T and MDCK-II. Allantoic fluid was harvested and checked for bacterial contamination. Sequences were checked by Sanger sequencing and using Geneious software. All experiments with infectious virus were conducted under BSL3-conditions.

### Polymerase assay

We investigated the mutation’s impact on the polymerase activity with a luciferase based minigenome assay as previously done [34]. Briefly, sub-confluent HEK293T cells were subjected to Lipofectamine 2000 based transfection of pHW plasmids containing the segments of the polymerase complex (polymerase basic protein 1 and 2 (PB1, PB2), polymerase acidic protein (PA) and nucleoprotein (NP)) as well H7-Nanoluciferase (NanoLuc) and Firefly-Luciferase. The latter was added as a transfection control. As negative control, the empty pHW vector was transfected instead of PB2. 48 h post-transfection cells were harvested using 2 × Lysis-Juice (PJK) and water 1:1. To enhance the signals, samples were frozen at −20 °C for at least 30 min. Subsequently, Firefly and NanoLuc reagent was added to the rethawed samples. Luminescence was detected with a GloMax^®^ Discover Microplate Reader. The NanoLuc expression was normalized against Firefly expression. Several attempts to transfect DF1 cells were not satisfactory due to the high background noise, therefore, we used standard HEK293 cells.

### Viral replication kinetics

The respective cells (CEK, DF-1, MDCK-II) were inoculated with virus suspension containing a multiplicity of infection (MOI) of 0.001. After 1 h of incubation at 37 °C, 5% CO2 the supernatant was removed. On MDCK-II and CEK cells all non-intracellular virus was inactivated using citric acid buffer (CBS, pH = 3.0). After washing thrice with PBS, MEM containing bovine serum albumin (BSA) was added. DF-1 cells detached after CBS treatment, therefore the extracellular virus was removed only by several washing steps with PBS. Subsequently, media consisting of MEM (Hank’s salts), MEM (Earle’s salts), NEA and 0.56% BSA solution (35%) was added. The cells were incubated at 37 °C. At the respective time points (1, 8, 24 and 48 h post-infection (hpi)) cells were collected using a cell scraper and immediately frozen at −70 °C for later titration.

### Virus titration

For virus titration confluent MDCK-II cells were infected in ten-fold dilutions with 250 µL viral suspension from either replication kinetics or swab samples. After incubation (1 h, 37 °C) and washing plaque test media containing Bacto-Agar was added. For titration of swab samples, medium containing 1% Penicillin/Streptomycin to counteract bacterial infections was used. Four days post-infection (dpi) the cells were fixed by applying crystal violet in formalin (0.1%) for 24 h. Subsequently the staining solution as well as the agarose was removed. Dried plates were counted and titres were calculated: Plaque forming units (PFU)/mL = PFU × dilution factor.

### Plaque size assay

To investigate cell-to-cell spread we infected confluent MDCK-II cells and subsequently, a standard plaque test (see above) was performed. After fixation, the cells were stained a second time. A total of 55 PFU (wt), and 87 PFU (ΔF2) were counted using a Nikon eclipse Ti as well as the corresponding software’s 3-point measurement option (NIS Elements BR).

### Flow cytometrical analysis of DF-1 cells

Confluent DF-1 cells were infected at MOI 0.1. After incubation (1 h, 37 °C, 5% CO_2_) the cells were washed once with PBS. MEM containing BSA was added, subsequently. 24 hpi the cells were trypsinized, washed and resuspended (PBS). Staining against surface antigens was performed in various 20 min intervals including washing steps with Cell staining buffer (PBS with 0.1% FBS, 0.4% Natrium acid (25% solution)). The cells were fixed and permeabilized with intracellular staining buffer (Biolegend) according to the manufacturer’s instructions. Subsequently, intracellular staining was performed.

Antibodies against surface antigens were diluted in Cell staining buffer, those against intracellular antigens in PermWash (Biolegend). Measurement was performed using the BD LSRFortessa™ Cell analyzer. Compensation and interpretation were done using BD FACSDIVA™ software.

### Animals and eggs

SPF chicken eggs used for virus isolation and propagation and animal experiment were sourced from Valo BioMedia (VALO SPF chickens). Eggs used in in vivo studies were hatched at FLI, Celle. Day old chicks were transferred to and raised at the animal facilities at FLI, Riems in a barn system. They were fed with commercial chicken feed (Panto KAK 2 mm).

### Antibodies

#### Primary antibodies

All primary antibodies are indicated in Table 1 (Primary antibodies used for multiparametric staining for flow cytometry).

**Table 1:** **Primary antibodies used for multiparametric staining for flow cytometry**

Antigen	Clone	Conjugate	Manufacturer	Dilution
AIV NP	HB65		In house	
Aqua Zombie		V500	Biolegend	
Chicken Bu-1	AV20	BV421	Southern Biotech	2 µg/mL
Chicken CD25	AV142	pure	Bio-Rad AbD serotec	1 µg/mL
Chicken CD3	CT-3	BV421	Southern Biotech	5 µg/mL
Chicken CD4	Feb 35	Pure	serotec	1 µg/mL
Chicken CD45	LT40	APC	Southern Biotech	1 µg/mL
Chicken CD8α	CT-8	Biotin	Southern Biotech	5 µg/mL
Chicken CD8β	EP42	pure	Southern Biotech	0.625 µg/mL
Chicken γδ TCR	TCR-1	FITC	Southern Biotech	5 µg/mL
Chicken KUL-01	KUL01	Biotin	Southern Biotech	5 µg/mL
Chicken MHC-II	2G11	PE	Southern Biotech	2 µg/mL
Mouse aCas3	C92-605	PE	BD	

#### Secondary antibodies

All secondary antibodies are indicated in Table 2 (Secondary antibodies used for multiparametric staining for flow cytometry).

**Table 2:** **Secondary antibodies used for multiparametric staining for flow cytometry**

Antigen	Clone	Conjugate	Manufacturer	Dilution
Mouse IgG1	A85-1	BUV650	BD	1:1000
Mouse IgG2α		PerCP	dianova	1:100
Mouse IgG2β		PE-Cy7	Southern Biotech	1:400
STAV		BV711	Biolegend	1:200

### Chicken experiment

All experiments were performed in accordance with the German regulations for animal welfare. The authorized ethics committee of the State Office of Agriculture, Food Safety and Fishery in Mecklenburg-Western Pomerania approved the conduction of the animal experiment (file no. 7221.3–1-060/17). Furthermore, the in vivo studies had the approval of the commissioner for animal welfare at the FLI, representing the Institutional Animal Care and Use Committee (IACUC).

Ten 7-week-old chickens per group were infected oculonasally with 200 µL of virus solution containing 10^5^ PFU/mL of the respective virus (wt or ΔF2) per animal. 24 hpi contact birds (*n* = 5) were introduced. Four dpi 3 animals per group were sacrificed for necropsy, organ samples were stored both in formaldehyde (histopathology) and at −70 °C.

Swab samples were collected from the oropharynx and cloaca, shaken and then stored at −70 °C respectively, in 1 mL swab sample medium (cell culture media containing 1% Enrofloxacin, 0.5% Lincomycin and 0.1% Gentamicin) at 1, 2, 3, 4, 7 and 10 dpi. To analyse the cellular immune response blood was collected at 1, 2, 4 and 7 dpi from 6 animals per group. If less than six birds, that were originally infected, survived, the number of samples was reduced accordingly. Contact birds were not considered for blood drawing.

The animals were scored daily as previously done [31]. Clinical signs were scored from 0 to 3. “0” represents a healthy animal, “1” is an animal with mild disease, exhibiting only one symptom. Animals with severe disease and/or multiple symptoms are scored as “2”, dead animals as “3”. Chickens, that were unable to move, feed or hydrate adequately and independently, were euthanized and scored as “3” the following day. Surviving chickens were euthanized 10 dpi using Isoflurane CP^®^ (CP pharma). Blood was drawn and plasma collected to test for seroconversion.

### Blood processing and staining for flow cytometry

Approximately 0.5 mL of blood were taken per chicken in lithium-heparin vials. After collecting and storing of plasma at −70 °C, the remaining blood cells were resuspended in PBS up to the original volume and then subjected to density centrifugation (900 × *g*, 30 min, without break) over a FICOLL-gradient. The buffy coat was collected, washed once and resuspended in PBS.

The isolated chicken PBMCs were divided and subsequently stained following a T cell panel or staining for antigen presenting cells. Between each staining step, cells were washed with Cell staining buffer. After the extracellular staining was complete the cells were fixed using Fixation buffer (BioLegend) according to the manufacturer’s protocol. The samples were measured using the BD LSRFortessa™ Cell analyzer. Compensation and interpretation were done using BD FACSDIVA™ software.

### Seroconversion

To determine seroconversion collected plasma was inactivated (56 °C, 120 min) and then tested for AIV nucleoprotein (NP) antibodies using commercially available enzyme-linked immunosorbent assay (ELISA) according to the manufacturer’s instructions (ID screen Influenza A Antibody Competition Multispecies kit, IDvet). Optical density (OD) was measured in a Tecan^®^ ELISA reader. In compliance with the manufacturer’s guidelines the cut-off point was 55%, samples lower than 45% were considered negative. Samples between 45 and 55% were considered as questionable.

### RNA extraction

100 mg per thawed organ sample was subjected to RNA extraction. After adding one steel bead (5 mm) and 1 mL Trizol per sample, the organs were homogenized in a Tissue Lyser II (Qiagen). Subsequently, RNA was extracted using the RNeasy kit (Qiagen) and stored at −70 °C.

### Virus load in organs

The viral copy numbers in organ samples were determined by generic matrix protein 1 (M1) real-time reverse-transcription PCR (RT-qPCR) using the SuperScript™ III One-Step RT-PCR System with Platinum™ Taq DNA Polymerase. Standard curves of H7N7 were included in the run, allowing the calculation of virus content in PFU/g equivalents by plotting the Ct-values of the standard curves. The utilized primers are supplied in Table 3 (Primers for M1 real-time RT-qPCR) and have been previously published [35].

**Table 3:** **Primers for M1 real-time RT-qPCR**

Primer	Sequence
IAV M1-rev	tgcaaaaacatcttcaagtytctg
IAV M1-for	agatgagtcttctaaccgaggtcg
IVA-M1 FAM	FAM-tcaggccccctcaaagccga-BHQ1

### Histopathological and immunohistochemical examination

Full autopsy was performed on all animals under BSL3 conditions. Samples from skin (wattle), nasal conchae, lung, heart, liver, pancreas, duodenum, cecum, brain, spleen, cecal tonsil, thymus, and bursa of Fabricius, of three birds at 4 dpi were fixed in 10% neutral buffered formalin. Tissues were paraffin-embedded and 2–3-μm-thick sections were stained with haematoxylin and eosin (HE). Immunohistochemistry (IHC) was performed for viral antigen detection using a primary antibody against the matrix (M1) protein of IAV (ATCC clone HB-64), the avidin and biotinylated enzyme (ABC) method, AEC (3-Amino-9-Ethylcarbazole) chromogen (red), and haematoxylin (blue) counterstain as described earlier [36]. Stained tissue slides were scanned using Hamamatsu S60 scanner and evaluated using NDPview.2 plus software (Version 2.8.24, Hamamatsu Photonics, K.K. Japan). Evaluation and interpretation were performed by a board-certified pathologist (AB, DiplECVP) in a blinded fashion. Archived tissue section from age-matched, non-infected chicken were included for comparison as controls. The severity of necrotizing inflammation was evaluated for the skin, nasal conchae, lung, heart, liver, pancreas, duodenum, cecum, brain. Additionally, for the brain, the area affected by perivascular inflammatory infiltrates and microglial cell nodules was recorded. The lesions and distribution of virus antigen was graded on an ordinal scale with scores 0 = no lesion/antigen, 1 = focal/rare, affected cells/tissue < 5% or up to 3 foci per tissue; 2 = multifocal, 6%–40% affected; 3 = coalescing, 41%–80% affected; 4 = diffuse, > 80% affected. To assess a possible induction of apoptosis we graded (i) the lymphocyte cellularity for the thymus, spleen, cecal tonsil and bursa; and (ii) the occurrence of tingible body macrophages (TBM, macrophages containing phagocytized, apoptotic cells) for the thymus. Decreased lymphocyte cellularity, increased TBM numbers was scored 0 = not present, 1 = minimal, 2 = mild, 3 = moderate, 4 = severe.

### Statistics

Statistical analyses were performed using GraphPad Prism 9. Data was tested for normality (Shapiro–Wilk test and Kolmogorov–Smirnov-test). Based on this, appropriate statistical tests were selected: For polymerase activity and DF-1 phenotype One-Way ANOVA was chosen. Data concerning replication kinetics, DF-1 phenotype at multiple MOI, clinical scoring, virus shedding, and cellular immunity were analysed by two-way ANOVA. Cell-to-cell-spread was calculated by Mann Whitney test. Survival rates were plotted as Kaplan Meier curves with Log-rank (Mantel-Cox) test. Significant differences were depicted according to the *P*-value as non-significant (ns) (*P* > 0.05), * (*P* ≤ 0.05), ** (*P* ≤ 0.01), *** (*P* ≤ 0.001), **** (*P* ≤ 0.0001).

## Results

### Deletion of PB1-F2 accelerates viral replication at early time points

**Figure 1:**
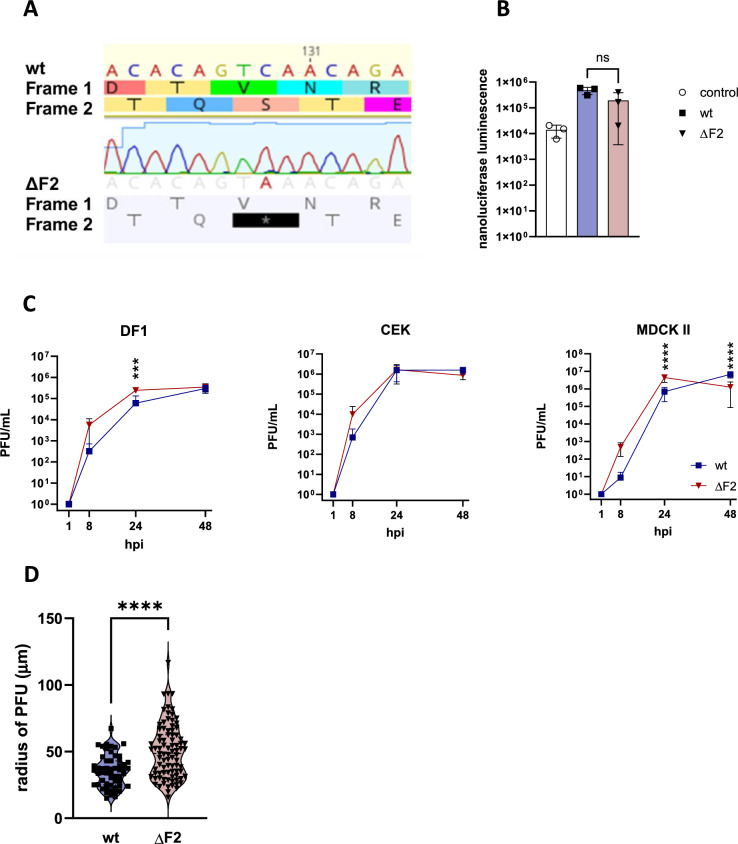
**In vitro characterization of wt and ΔF2. A** The mutation (C138A) introduced a stop codon to the PB1-F2 reading frame, while the PB1 frame remains intact. **B** HEK293T cells were transfected with plasmids of the polymerase complex (PB1, PB2, PA, NP) as well as Nanoluciferase and Firefly Luciferase (as transfection control). 48 hpi luminescence of lysed cells was analysed and normalized against transfection control. The data is shown as mean (SD), control (white), wt (blue), ΔF2 (red), statistical analysis was done by One-Way ANOVA with Tukey’s multiple comparisons test. The data is representative of one of three separate experiments. **C** Viral replication kinetics were performed using chicken fibroblasts (DF-1), chicken embryo kidney cells (CEK) and Madin-Darby canine kidney cells (MDCK-II). Cells were infected at a multiplicity of infection (MOI) of 0.001, collected and titrated by Plaque test. Pooled data from three separate experiments is depicted as mean (SD), statistics are calculated by two-way ANOVA.** D** To assess cell-to-cell spread at least 50 PFU per virus were measured. Results are depicted as mean (SD), statistical analysis was performed using the Mann Whitney test (*p* < 0.0001).

We constructed two recombinant viruses based on A/chicken/Germany/AR1385/2015 (AR1385). This virus was isolated in 2015 from a limited outbreak in flocks of laying hens in Emsland, Germany [30]. Both wt and ΔF2 (Figure 1A) viruses replicated to similar titres (1–2 × 10^5^ plague forming units (PFU)/mL viral stock) in embryonated chicken eggs. Sequencing of stock viruses (HA, NA, PB1) revealed no off-target mutations.

The IAV polymerase complex is composed of four subunits (PB2, PB1, PA, and NP). These segments make up the minireplicon of viral RNA transcription and replication and can be analysed in a transfection-based polymerase activity assay. To investigate whether the introduced mutation in PB1-F2, which is encoded on the PB1 segment, affects overall polymerase activity we conducted minigenome reporter assays. After transfection of HEK293T cells with the aforementioned minireplicon segments as well as H7-Nanoluciferase (NanoLuc) and Firefly-Luciferase, NanoLuc luminescence was measured and normalized to the luminescence of the transfection control (Firefly Luciferase). Both wt and ΔF2 were able to replicate the luciferase reporter genome as was demonstrated when compared to the control, which lacked the PB2 component of the viral polymerase (Figure 1B). However, while the results of ΔF2 varied more than those of wt, no significant impact on the polymerase activity was observed.

To assess viral fitness, we performed viral replication kinetics in an avian (chicken fibroblasts (DF-1)) cell line, primary chicken embryo kidney cells (CEK), and a permissive mammalian cell line (Madin-Darby canine kidney cells type-II (MDCK-II)) (Figure 1C). ΔF2 replicated significantly better than wt 24 h post-infection (hpi) in DF-1 (4.1 × better) and MDCK-II cells (6.4 × better). However, this effect was compensated (DF-1) or reversed (MDCK-II) 48 hpi. In the latter, wt grew to significantly higher titres than ΔF2 (5.3 × higher). However, since the difference is less than one log-fold it is questionable whether these effects are of biological relevance. In all cells there was a trend, although not significant, for faster replication of ΔF2 8 hpi. To study the effect of PB1-F2 on cell-to-cell spread, we measured plaque diameters induced by wt and ΔF2 (Figure 1D). In this study, the average plaques formed after ΔF2 inoculation were found to be 14.7 µm larger in diameter compared to the wt plaques. Consequently, the knockout of PB1-F2 resulted in a more efficient establishment of infection in neighbouring MDCK-II cells. It is important to note, however, that the knockout virus again exhibited a higher degree of variability.

### PB1-F2 limits apoptosis in infected cells

**Figure 2:**
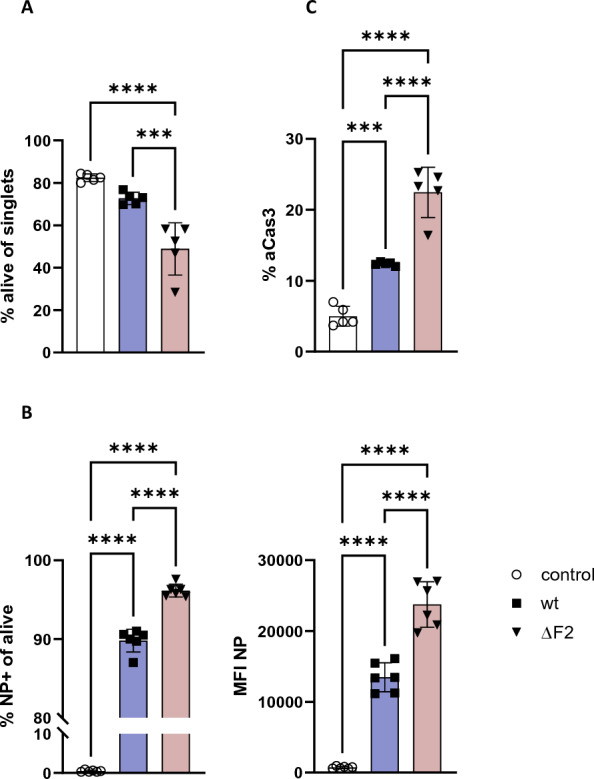
**Phenotype of infected DF-1 cells.** All experiments were conducted using DF-1 cells infected at MOI 0.1 and harvested 24 hpi. The cells were stained and analysed by flow cytometry. Data is shown as mean (SD) control (white), wt (blue), ΔF2 (red). Statistical analysis was calculated as One-Way ANOVA. Depicted are representative results from one of at least three individual experiments. **A** Live-dead-staining was performed with Zombie Aqua™ viability dye. Zombie-negative fraction of a population of single cells. (*p* < 0.0001) **B** Live cells were further characterized using a monoclonal antibody against nucleoprotein (NP) to stain infected cells. NP positive cells (*p* < 0.0001) as well as the expression of NP in the cells (as MFI NP, *p* < 0.0001). **C** Apoptotic cells were identified based on positivity for active Caspase 3 (*p* < 0.0001). Caspase expression was analysed in Zombie-negative, NP-positive cells. A population of naïve, Zombie-negative cells was used as control.

To investigate effects of AIV full length PB1-F2 on cellular viability, we employed flow cytometry. First, we established the infection dose in titration experiments with DF-1 cells using multiplicities of infection (MOI) of 0.1, 0.01, and 0.001 and collecting the cells for staining 24 hpi (Additional file 1). Applying MOI 0.1, a large population of infected IAV nucleoprotein (NP)-positive cells were detected (wt: 35.9% of live cells) and viability was only slightly reduced (wt: 72.7% of total cells). Therefore, all following flow cytometry experiments were conducted at MOI 0.1. When repeating our experiments to include staining for apoptotic cells (Additional file 2), we reached even higher infection rates (wt: 89.8%, ΔF2: 96.2%), while the differences between both viruses remained significant. Contrary to pro-apoptotic effects reported for full-length PB1-F2 [13, 37], we observed that PB1-F2 appeared to restrict cell death in avian cells. While 73% of the harvested cells survived wt infection, only 49% of ΔF2-infected cells were viable 24 hpi (Figure 2A). Considering the viral replication kinetics (Figure 1C), we assume that the early differences in viral titres resulted from variations in cell viability. However, the compensation or reversal in replication patterns observed 48 hpi might have been caused by different survival rates of infected cells.

We examined the infection status of the viable cells by staining for the viral NP and found wt infected 89.8% of cells, while ΔF2 was able to infect significantly more cells (96.2%) (Figure 2B). In addition, the mean fluorescent intensity for each NP-positive cell was measured and cells infected with ΔF2 had a 1.8 × higher intensity compared to wt, suggesting that upon infection more NP was present in cells infected by ΔF2 virus. Thus, PB1-F2 deletion spiked infection rates and also resulted in significantly increased synthesis of certain viral proteins (NP) in infected cells.

To evaluate whether the difference in viability was due to variability in apoptosis, we identified apoptotic cells by staining for active Caspase 3 (aCas3, Figure 2C). Both viruses caused significant apoptosis compared to the naïve control. In accordance with the live-dead staining, ΔF2 caused heightened apoptosis of infected cells (ΔF2: 22.5% vs. wt: 12.4%). Our data show that while infection generally triggers apoptosis in chicken cells, full length H7N7 PB1-F2, unlike the full-length versions carried by some mammal-adapted IAV [24], restricts apoptosis to a certain extent.

### Truncation of PB1-F2 increases survival in chickens

**Figure 3:**
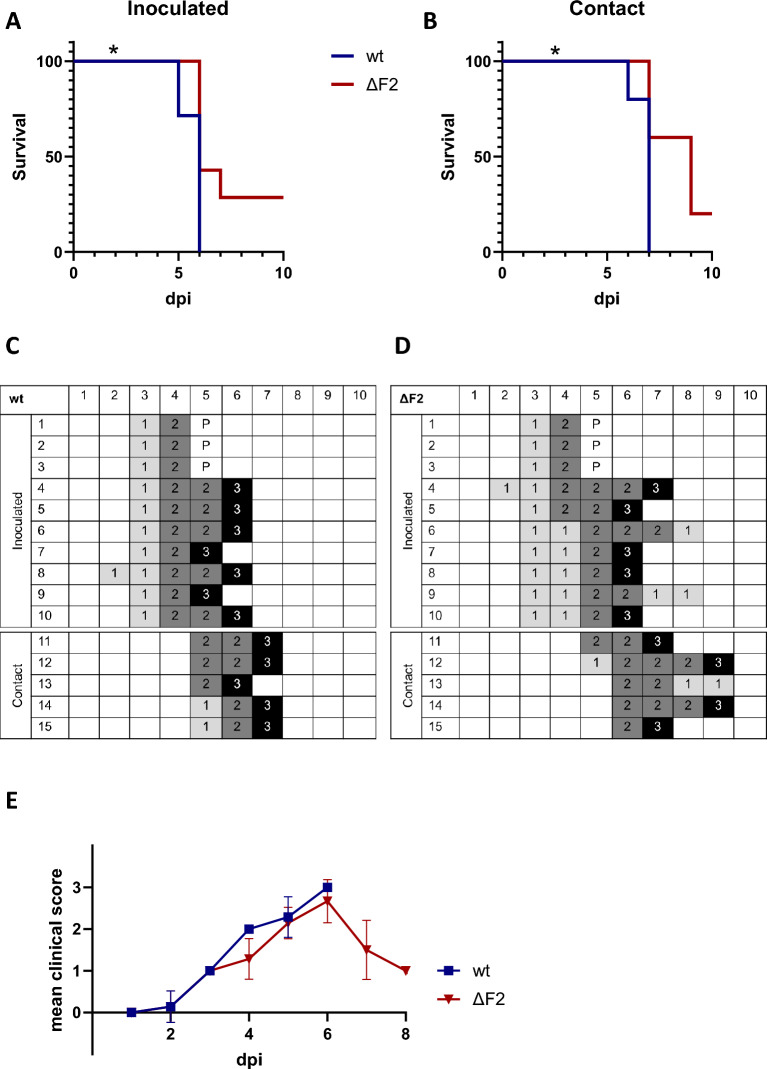
**Expression of PB1-F2 altered survival period and clinical onset in chickens. A, B** Kaplan Meier plotting of survival rates of inoculated (n = 7, **A**) and contact animals (*n* = 5, **B**), wt (blue), ΔF2 (red). Statistical analysis was done by Log-rank (Mantel-Cox) test (infected: *p* = 0.0287; contact: *p* = 0.0411) **C, D** Clinical scoring for inoculates (1–10) and contact birds (11–15) after infection with wt (C) or ΔF2 (D). Scores (0–3) were assigned as follows: 0: no symptoms, 1: one symptom, 2: multiple signs of disease, 3: death. 4 dpi 3 animals per group were sacrificed for necropsy and sampling (P). **E** Clinical scoring of chickens depicted as Mean with Standard deviation. Statistical analysis was performed as a two-way ANOVA with Šídák’s multiple comparisons test (*p* = 0.2850).

To study the impact of PB1-F2 length on virus fitness in chickens, we inoculated chickens via the oculonasal route. To investigate the virus transmission, we introduced sentinels to the inoculated chickens 1 dpi. All chickens were observed daily and the pathogenicity index (PI) was calculated as previously done [31]. After infection with either wt or ΔF2, all inoculated chickens showed severe signs of disease. All wt-infected chickens and their cohoused chickens died, while 5 of 7 ΔF2-infected and 4 of 5 contact birds died (Figures 3A and B). The PI for wt- and ΔF2-inoculated chickens was 2.0 and 1.6, respectively. The viability pattern is also reflected in the clinical scoring: the survivors (ΔF2 animals #6, #9 and #13) showed signs of disease but recovered from infection (Figure 3D). Overall, both groups started to show symptoms at similar time points, however clinical disease progressed faster in wt-infected chickens (Figure 3C) than in those inoculated with the knockout virus (Figure 3D). Even though not statistically significant, this trend is also evident when directly comparing the groups (Figure 3E). The signs of disease varied depending on the virus: wt caused severe diarrhoea, whereas ΔF2 caused neurological symptoms (headshaking, dyscoordination) in approximately 50% of the infected birds. The mentioned neurological symptoms were detected at late stages of the infection (5 dpi and later). A significant decrease of the survival rate in both inoculated (Figure 3A) and contact birds (Figure 3B) was observed when full-length PB1-F2 was expressed (wt). The medium time to death was 5.7 dpi for wt-infected chicken and 6.2 dpi for ΔF2-infected chickens.

Subsequently, we performed a NP antibody competition ELISA using plasma collected from the surviving chickens 10 dpi. All survivors seroconverted, confirming that they were indeed infected with the virus. Thus, deletion of PB1-F2 does increase chances of survival after successful infection with HPAIV H7N7.

### PB1-F2 enhances gastrointestinal shedding and viral replication in the gut

**Figure 4:**
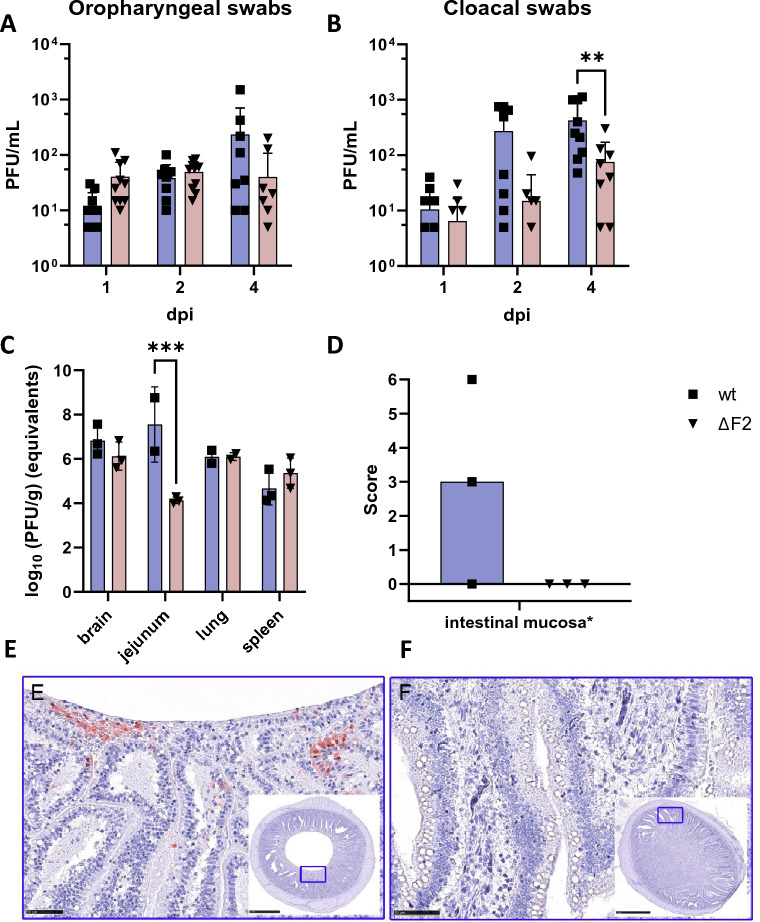
**PB1-F2 alters virus shedding. A**, **B** Oropharyngeal (**A**) and cloacal swabs (**B**) of wt (blue) and ΔF2 (red) animals were titrated by plaque assay. Statistical analysis was done by Two-Way ANOVA. **C** RNA was extracted from tissue samples of brain, jejunum, lung and spleen. Subsequently, real-time reverse-transcription PCR (RT-qPCR) was performed. Standard curves of H7N7 were included in the run, allowing the calculation of virus content in PFU/ml by plotting the Ct-values of the standard curves. Data is depicted as log (10) of PFU per gram of tissue (PFU/g) equivalents. For statistical analysis Two-Way ANOVA was performed. Virus antigen score for intestinal mucosa proves viral replication after wt but not after ΔF2 infection. Influenza virus matrix (M1) protein detection by IHC, blind scoring, dots represent individual scores, bar represents median group score. Scores given as no antigen = 0, rare/focal = 1, multifocal = 2, coalescing = 3, diffuse = 4 (**D**). Representative images for IHC-based detection of M1 protein in enterocytes after wt (**E**) but not after ΔF2 (**F**) infection. IHC, using avidin and biotinylated enzyme method, 3-Amino-9-Ethylcarbazole chromogen (red), and haematoxylin (blue) counterstain. Bar 50 µm, inlay with a cross section of the small intestine and rectangle showing the selected representative image section.

During the animal experiment, we collected oropharyngeal and cloacal swab samples to determine viral shedding 1, 2, 3, and 4 dpi. Titration by plaque assay revealed no significant difference between the two groups and timepoints in shedding from the oropharyngeal cavity (Figure 4A). The cloacal shedding, however, was significantly impacted by expression of PB1-F2. 4 dpi wt-infected chickens on average shed 10^2.63^ plaque forming units per mL (PFU/mL), compared to ΔF2’s 10^1.88^ PFU/mL. While 1 dpi swab samples showed no difference with respect to cloacal shedding, samples collected 2 dpi were indicative of cloacal shedding in wt-infected chickens: wt 10^2.44^ PFU/ml vs. ΔF2 10^1.18^ PFU/mL. In line with these observations, viral antigen detection in the intestine was restricted to wt-infected chicken (Figures 4D–F). We confirmed that infection with wt virus resulted in more abundant M1 copy number in the jejunum (Figure 4C). This was in line with reported gastrointestinal symptoms in wt-infected chickens.

### PB1-F2 has no effect on systemic tissue tropism and morphological lesion development but may influence the death of immune cells in vivo

**Figure 5:**
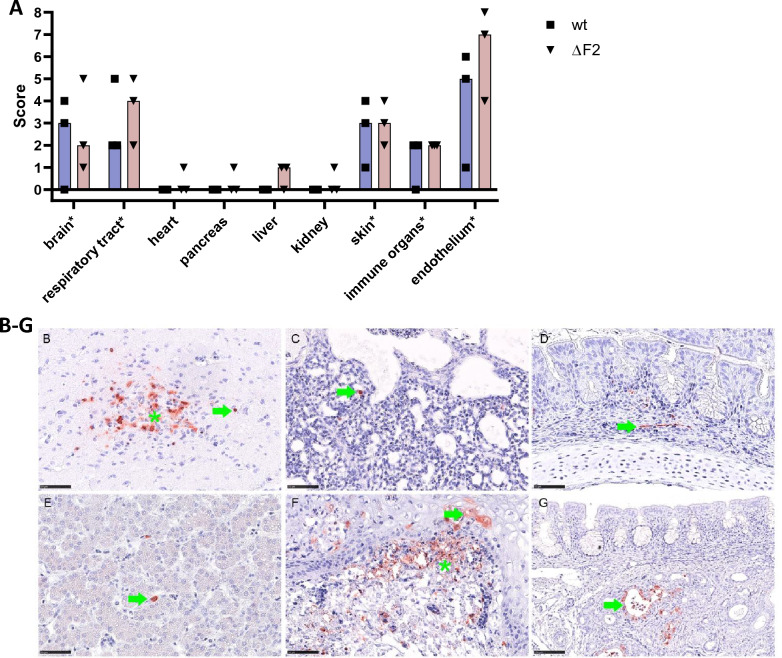
**PB1-F2 does not alter the systemic viral tissue spread.** By means of IHC for IAV M1, ΔF2 and wild type infection yielded high virus antigen scores in the brain, respiratory tract, skin and immune organs. Both viruses exhibited strong endotheliotropism**.** IHC, blind scoring, dots represent individual scores, bar represents median group score. Scores given as no antigen = 0, rare/focal = 1, multifocal = 2, coalescing = 3, diffuse = 4. Asterisks indicate cumulative scores for the brain (for neurons, glial cells, ependymal cells), respiratory tract (respiratory and glandular epithelium of conchae and lung), skin (epidermal and mesenchymal cells), immune organs (immune and mesenchymal cells of thymus, spleen and bursa), and endothelium (all organs tested) (**A**). Representative images for IHC-based detection of M protein in target cell indicating neurons (asterisk) and glial cells (arrow) in the brain (**B**), air capillaries in the lung (arrow) (**C**), endothelium (arrow) exemplarily shown in the nasal conchae (**D**), sinusoid lining cells (arrow) of the liver (**E**), epidermal epithelium (arrow) and dermal mesenchymal cells (asterisk) in the skin (**F**) and glands (arrow) in the nasal conchae (**G**). **B**–**G** IHC, using avidin and biotinylated enzyme method, 3-Amino-9-Ethylcarbazole chromogen (red), and haematoxylin (blue) counterstain. Bar 50 µm (**B**–**F**), 100 µm (**G**).

Supporting the data on PB1-F2-mediated enhanced gastrointestinal shedding, presence of viral antigen detection in the intestine was restricted to wt-infected chicken (Figures 4D–F). However, no significant difference was found for the remaining tissues tested, including the brain, the respiratory tract, heart, pancreas, liver, kidney, skin, and immune organs. Both, ΔF2 and wt infection led to high virus antigen scores in the brain, respiratory tract, skin, and immune organs. Both viruses exhibited endotheliotropism (Figure 5A). Target cells included the glandular and respiratory epithelium in the nasal mucosa, neurons, glial cells, and ependymal cells in the brain, air capillary cells in the lung, cardiomyocytes in the heart, acinar cells in the pancreas, sinusoid lining cells in the liver, tubular epithelium in the kidney, epidermal and mesenchymal cells (not specified) in the skin, as well as immune and mesenchymal cells in immune organs (Figures 5B–G). Of note, the endotheliotropism was particularly prominent in the dermis (wattle, Figure 5D) and mucosa of the nasal conchae.

**Figure 6:**
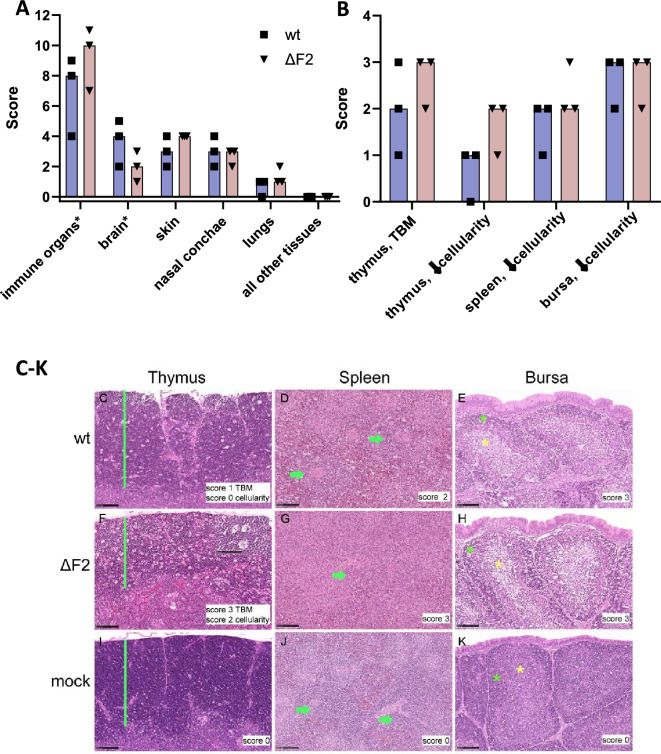
**PB1-F2 may influence apoptosis induction in immune organs but has no impact on the development of necrotizing inflammation in other tissues.** Scoring of tissue lesions after HE staining yielded comparable results after ΔF2 and wt infection in most tissues including the brain, skin, nasal conchae, and lungs. Particularly high lesion scores were found in immune organs after ΔF2 infection (**A**). Detailed scoring of immune organs after HE staining showing particular high lesion scores in the thymus after ΔF2 infection (**B**). Scores for necrotizing inflammation given as 0 = no lesion, 1 = rare, affected cells/tissue < 5% or up to 3 foci per tissue; 2 = multifocal, 6–40% affected; 3 = coalescing, 41%–80% affected; 4 = diffuse, > 80% affected. Decreased lymphocyte cellularity, increased tingible body macrophages (TBM) numbers was scored 0 = not present, 1 = minimal, 2 = mild, 3 = moderate, 4 = severe. Asterisks indicate cumulative scores for the immune organs (thymus, spleen, bursa) and brain (necrotizing inflammation, glial nodules, perivascular infiltrates). Representative images for HE-based lesion scores after ΔF2 or wt infection in the thymus, spleen and bursa (**C**–**K**). Green bar = cortex of the thymus with inlay in (**F**) showing TBM; green arrow = periarteriolar lymphoid sheaths in the spleen; green asterisk = cortex of the bursa, yellow asterisk = medulla of the bursa. Hematoxylin and eosin staining, bar 100 µm.

Histopathology did not identify significant differences between chickens after wt or ΔF2 infection (Figure 6A). For both groups, a necrotizing inflammation was found in the brain, skin, nasal conchae and lungs. The detailed analysis of immune tissues yielded particularly high lesion scores for ΔF2-infected chickens in the thymus, showing decreased lymphocyte cellularity and increased tingible body macrophages (TBM) numbers and lymphocyte apoptosis/necrosis but not in the spleen and bursa (Figures 6B–K).

### PB1-F2 marginally influences dynamics of blood immune cells

**Figure 7:**
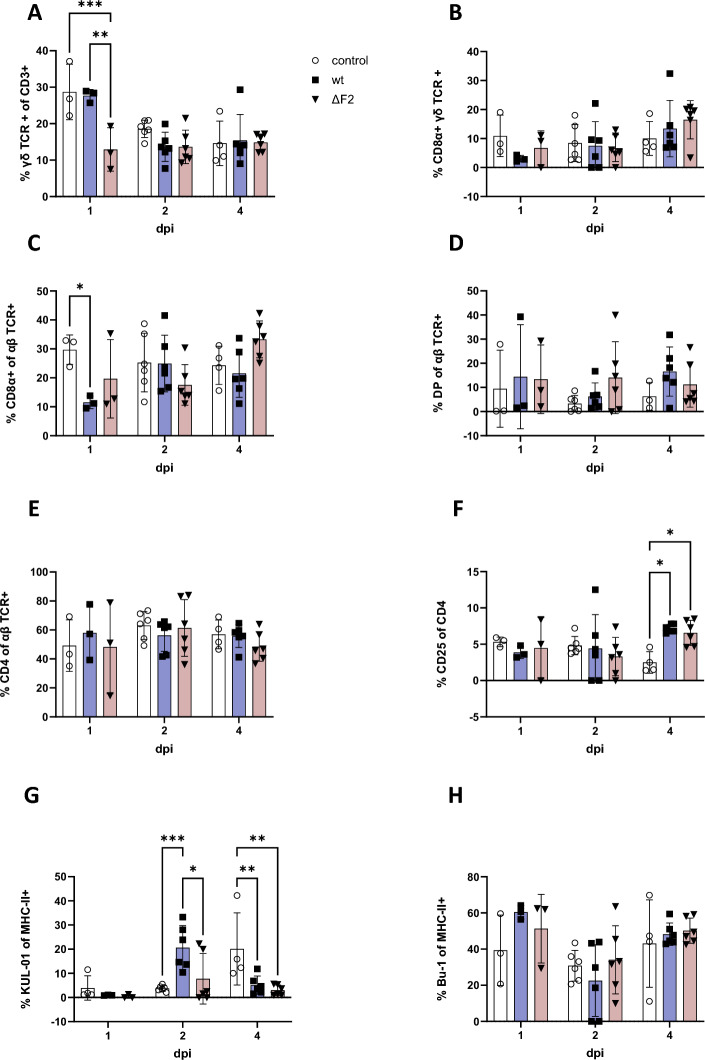
**PB1-F2 marginally affects frequencies of leukocyte subsets.** After ex vivo isolation leukocytes were stained for multiparametric flow cytometry. Single, live T cells (CD3 + , **A**–**F**) or single, live antigen presenting cells (APC, CD4−, MHC-II+ , **G**–**H**) were phenotyped. **A** Frequencies of γδ T cells, **B** Activation (CD8α +) of γδ T cells, **C** Frequencies of Effector T cells (CD8 +) from αβ T cell fraction, **D** CD4 + CD8 + αβ T cells, **E** T helper cells (CD4 +) from αβ T cell fraction, **F** percentage of activated (CD25 +) T helper cells, **G** Monocyte (KUL-01 +) fraction of APC, **H** Fraction of B cells (Bu-1 +) of APC. Statistical analysis was performed as two-way ANOVA with Tukey’s multiple comparisons test. Data is shown as mean (SD), control (white), wt (blue), ΔF2 (red).

Since the impact of PB1-F2 on virulence could be caused by immune-driven inflammation, we analysed blood-derived T-lymphocytes (Figures 7A–F) and antigen-presenting cells (Figures 7G–H) by multiparametric flow cytometry. We observed few significant differences between wt- and ΔF2-infected chickens: 1 dpi animals inoculated with ΔF2 showed lower frequencies of γδ T lymphocytes (Figure 7A, wt: 27.7% vs. ΔF2: 12.9%)), which have features of both innate and adaptive immunity [38]. Nevertheless, the activation status of γδ T cells, detected by expression of CD8, was not affected compared to the naïve control (Figure 7B). Additionally, both viruses caused a lower frequencies of T effector cells 1 dpi (Figure 7C) and an increase in activated T helper cells (Figure 7F) 4 dpi compared to the naïve control group.

Furthermore, the percentage of monocytes (KUL-01 + MHCII + antigen presenting cells) was elevated in wt-infected chicken 2 dpi (20.7%) compared to ΔF2-infected chickens (7.7%). However, these monocyte frequencies were similarly low in both groups 4 dpi (wt: 5.1%, ΔF2: 3.0% vs. naïve control: 20.1%). Overall, the observed effects were only temporary.

## Discussion

The aim of this study was to provide information on the relevance of PB1-F2 after infection of chickens with H7 IAV. Data on the effects of PB1-F2 in avian hosts are limited and contradictory.

Our studies showed that the PB1-F2 knockout enhanced viral replication in vitro accompanied by increased infection rates and synthesis of viral proteins, as well as ameliorated cell-to-cell spread. Potentially, due to antagonistic interaction with the cellular antiviral mechanisms [23], 48 hpi the replication advantage was either compensated or completely reversed, presumably due to increased survival rates of wt-infected cells, allowing efficient replication. However, due to the transiency of effects and the low log-fold difference, the biological impact might be limited. By contrast, expression of PB1-F2 is largely described to not influence viral titres in vitro (H9N2 A/chicken/Pakistan/UDL01/08 (UDL01) [25], PR8 [19]), though it should be noted that different MOIs were used (0.0001 [25] or 1 [19]). However, PB1-F2-containing H1N1 strains (PR8, A/Beijing/11/56 PB1-F2, and A/Brevig Mission/1/18 PB1-F2 in PR8 backbone) were reported to replicate to higher titres than the viruses lacking full-length PB1-F2 in a dose-dependent manner [39]. Consistent with our data, viruses replicating to higher titres also formed larger plaques and this effect was also exclusively present at early time points. [39]. This suggests that full-length PB1-F2 of mammalian- and avian-adapted viruses have opposing effects on viral replication.

Since survival of infected cells contributes to efficient viral replication, we investigated the viability of infected DF-1 cells. Surprisingly, we found that expression of full-length PB1-F2 increased the survival rate of infected chicken fibroblasts by limiting apoptosis. In contrast, PB1-F2 is largely considered to be pro-apoptotic in mammalian cells but its effects vary depending on virus strain and mammalian host cell. Indeed, LPAIV H6N1 (A/turkey/England/198/09), LPAIV H2N3 (A/mallard duck/England/7277/06) and recombinant HPAIV H5N1 demonstrated pro-apoptotic function in infected porcine macrophages, while several H1N1 viruses showed no effect in the same experimental setting [37]. Furthermore, PR8 PB1-F2 is pro-apoptotic [24, 40], whereas reports on PB1-F2 of 1918 H1N1 are diverse: PB1-F2 of A/Brevig Mission/1/18 in a PR8 backbone did not enhance apoptosis [24], while using the same ORF in the backbone of USSR/90/77 increased apoptosis compared to a knockout variant in murine macrophages [41]. HPAIV H7N7 do not share cytotoxic motifs (I68, L69, V70) previously described for PB1-F2 (Additional files 3, 4, 5) [42], which could be one reason why apoptotic degradation was limited. However, since all previous data was gathered from mammalian cell culture systems, it is plausible to consider that the restriction of apoptosis, despite the high conservation rate of apoptotic pathways [43], may be an exclusive feature of avian cells.

To investigate the recombinant H7N7 viruses in a complex organism and to explore potential tissue tropism and effects on cellular immunity, we infected White Leghorn SPF chickens. Interestingly, the truncation of PB1-F2 increased chances of survival of both inoculated and cohoused birds. While there were no differences in infection rates and disease onset, the ΔF2-infected chickens showed slower disease progression and some even recovered. Opposing effects were described for chickens infected with HPAI H5N1 (A/duck/Niger/2090/2006 (Nig06)) [26] and LPAIV H9N2 (A/chicken/Pakistan/UDL01/08) [25].

In other avian species the effect of PB1-F2 appears to be similarly variable: Swine influenza virus H3N2 (A/swine/Minnesota/1145/2007) lacking PB1-F2 infected turkeys more efficiently than its full-length counterparts [44]. However, while chickens and turkeys are both susceptible to AIV infection [45–47], it is not reasonable to expect swine-adapted and avian-adapted viruses to cause similar effects. Only the inoculation of ducks with HPAI H5N1 (VN/1203/04) generated results consistent with our obtained data: Animals infected with the knockout virus showed delayed onset of clinical disease and systemic spread of the virus [27]. In summary, the effect of PB1-F2 on the survival of infected birds depends on many variables.

Furthermore, our experiments showed that H7N7 HPAIV PB1-F2 promoted cloacal shedding, while its influence on oropharyngeal shedding was negligible. Conversely, LPAIV H9N2 prolongs buccal shedding [25]. Increased faecal virus shedding was associated with severe diarrhoea in wt-infected chickens, whereas ΔF2-infected chickens showed mainly neurological symptoms. Fittingly, the gastrointestinal symptoms were associated with increased viral copy numbers in the gut. The increased cloacal shedding as well as the gastrointestinal symptoms indicate that PB1-F2 facilitates gastrointestinal tissue tropism [48]. Immunohistochemistry supported this, as only wt-infected chickens showed viral antigen in the intestine, highlighting the severe viral burden in gastrointestinal tissues. However, histopathology did not reveal any lesions in the evaluated intestinal tissues (duodenum, cecum) to support the diarrhoea reported in wt-infected chickens. It cannot be excluded that lesions could have been detected in non-examined intestinal segments. While both viruses caused lesions of the central nervous system, the differences in clinical reaction might be due to different localizations, potentially caused by the deletion of PB1-F2.

Chickens of similar age inoculated with H5N1 (Nig06) virus showed similar viral shedding patterns: The group inoculated with Nig06 expressing PB1-F2 exhibited significantly higher viral copy numbers in the intestine and pancreas compared to the ΔF2 group [26].

Strikingly full-length PB1-F2 is conserved among many avian IAV isolates, however its prevalence varies depending on avian order. While only 87% of isolates from *Galliformes* carry full-length PB1-F2, isolates from *Anseriformes* (waterfowl) and *Charadriiformes* (waders, gulls, auks) express a complete PB1-F2 ORF at 97% and 98%, respectively [25]. This suggests that while full-length PB1-F2 is advantageous to chicken-adapted virus, it is even more crucial for viruses in the natural reservoir of waterfowl and shore birds. This might be explained by different modes of transmission, as AIV in wild aquatic birds replicate primarily in the gut and are excreted at high doses into the environment. Contact with contaminated surface water, either directly or indirectly, provides the main source of infection to susceptible waterfowl [3]. We hypothesize, that PB1-F2 ensures effective viral spread by causing gastrointestinal tropism in the natural reservoirs, explaining its high prevalence in these orders, specifically.

In addition, histopathology identified decreased lymphocyte cellularity in the thymus. Particularly high scores in the thymus were found after ΔF2 virus infection, accompanied by increased numbers of TBM containing phagocytized, apoptotic cells [49]. Increased lymphocyte apoptosis may result from direct thymic immunomodulatory effects or stress (e.g. disease-related decreased food intake, glucocorticoid release) [50]. Severe ongoing apoptosis results in greatly reduced lymphocyte cellularity. Increased TBM and decreased cellularity in the thymus but also in other immune organs may thus be a direct effect of immunomodulation or a secondary effect of stress. Either PB1-F2 or lack thereof [24] or death ligands (e.g. TNF, FasL or TRAIL), secreted due to severe inflammation [51], could contribute to apoptotic cell death.

We suggest that the immunomodulatory effect is directly virus-related. Our in vitro data demonstrated that both viruses caused apoptosis. While ΔF2 infection yielded more apoptotic DF-1 cells at the same infectious dose in vitro, the clinical data 4 dpi showed higher clinical scores for wt-infected birds compared to ΔF2-infected animals. Therefore, a higher level of disease-associated stress is to be expected in the wt-infected group, which in turn may outweigh ΔF2’s superior pro-apoptotic function.

The comparative data available was obtained in mammals. Here, the virulence of PB1-F2 containing viruses is partly attributed to its immunopathogenic effects: 1918 PB1-F2 in a PR8 backbone increased the cellularity of murine bronchoalveolar lavage fluid by an influx of T lymphocytes, macrophages, and neutrophils. This induced severe lung inflammation and lesions [24, 52], that in turn cause increased susceptibility to bacterial superinfections [21, 52]. Furthermore, PB1-F2 was shown to reduce the adaptive immune response due to its apoptotic properties, both directly, by causing apoptosis of T cells [53], as well as indirectly by decreasing antigen presentation by eliminating antigen presenting cells [13]. Surprisingly, HPAIV H7N7 PB1-F2 only marginally affected cellular immunity. We reported a significant reduction of γδ T lymphocytes in ΔF2-inoculated chickens 1 dpi. In mammals, they were reported to recognize antigens independently of MHC-II [38], and have antiviral properties [54]. In chickens, both effector and regulatory functions have been described [55–57]. Given the importance of γδ T cells at early stages of infection, it is likely that ΔF2 induces recruitment of γδ T cells into tissues. Additionally, wt-infected animals showed elevated monocyte levels 2 dpi. Overall, all reported effects were transient and had no discernible clinical impact.

It should be noted that our results showed a wide statistical spread. The reasons for this are twofold: firstly, genetic variance of the animals has an impact. Secondly, there were individual differences in disease onset and progression, which in turn caused variation in leukocyte levels and distribution. Furthermore, since HPAIV H7N7 induced severe disease in all infected animals, it is reasonable to assume, that disease progression in both wt and ΔF2 was too fast and grave to allow for distinct immunological profiles.

In conclusion, HPAIV H7N7 PB1-F2 facilitates viral adaptation in vitro by limiting apoptosis of infected chicken fibroblasts. Apart from replication deficits at early time points in vitro, we found no adverse effects of PB1-F2 in vitro. The virus expressing full-length PB1-F2 was more virulent and showed enhanced cloacal shedding and gastrointestinal tropism. Therefore, we propose that PB1-F2 is a virulence factor in chickens, that ensures effective viral spread.

### Supplementary Information

**Additional file 1: Dose finding studies for phenotyping of DF-1 cells**. Confluent DF-1 cells were infected at different MOI (0.1, 0.01, 0.001) and subsequently collected and stained for flow cytometrical analysis. **A.** After Life-dead staining with Zombie Aqua single, living cells were selected. Statistical analysis was done as two-way ANOVA with Tukey’s multiple comparison test. **B.** Living cells were further characterized with a monoclonal NP antibody. Statistical analyses were performed using two-way ANOVA with Šídák’s multiple comparisons test.

**Additional file 2: Representative Gating strategy for in vitro flow cytometrical phenotyping.** Analyses were performed on pre-gated singlets. After selecting the relevant cell population (SSC/FSC) Zombie-negative NP + (infected) or Zombie-negative NP-negative (naïve control) were tested for their aCas3 expression.

**Additional file 3: Protein sequence of AR1385 PB1-F2.** Highlighted in red is the insertion sight for the introduced mutation in ΔF2. The positions were cytotoxic motifs were previously published are marked grey, the motifs published by Alymova et al. [42] are supplied beneath. For detailed information fasta files are provided (Additional files 4 and 5).

**Additional file 4.** **FASTA file H7N7 AR 1385_15 wt PB1-F2**.

**Additional file 5.** **FASTA file H7N7 AR 1385_15 delta F2 PB1-F2**.
