# Supplementary figures and images for: The role of PB1-F2 in adaptation of high pathogenicity avian influenza virus H7N7 in chickens
Source: Vet Res. 2024 Jan 3;55:5. doi: 10.1186/s13567-023-01257-8 (PMC10765749; doi:10.1186/s13567-023-01257-8)

## Slide 1
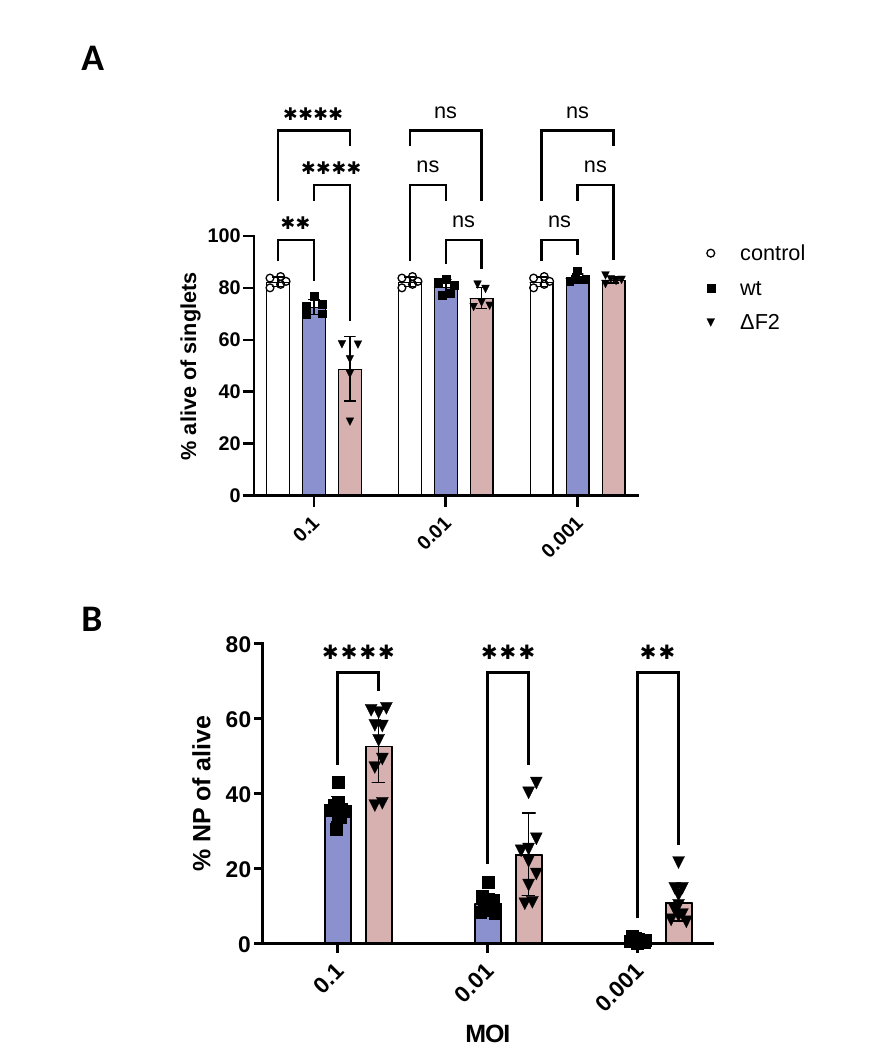

A
B

Supplement: Supplementary file 1 — Additional file 1: Dose finding studies for phenotyping of DF-1 cells. Confluent DF-1 cells were infected at different MOI (0.1, 0.01, 0.001) and subsequently collected and stained for flow cytometrical analysis. A. After Life-dead staining with Zombie Aqua single, living cells were selected. Statistical analysis was done as two-way ANOVA with Tukey’s multiple comparison test. B. Living cells were further characterized with a monoclonal NP antibody. Statistical analyses were performed using two-way ANOVA with Šídák’s multiple comparisons test. [file 13567_2023_1257_MOESM1_ESM.pptx]

## Slide 1
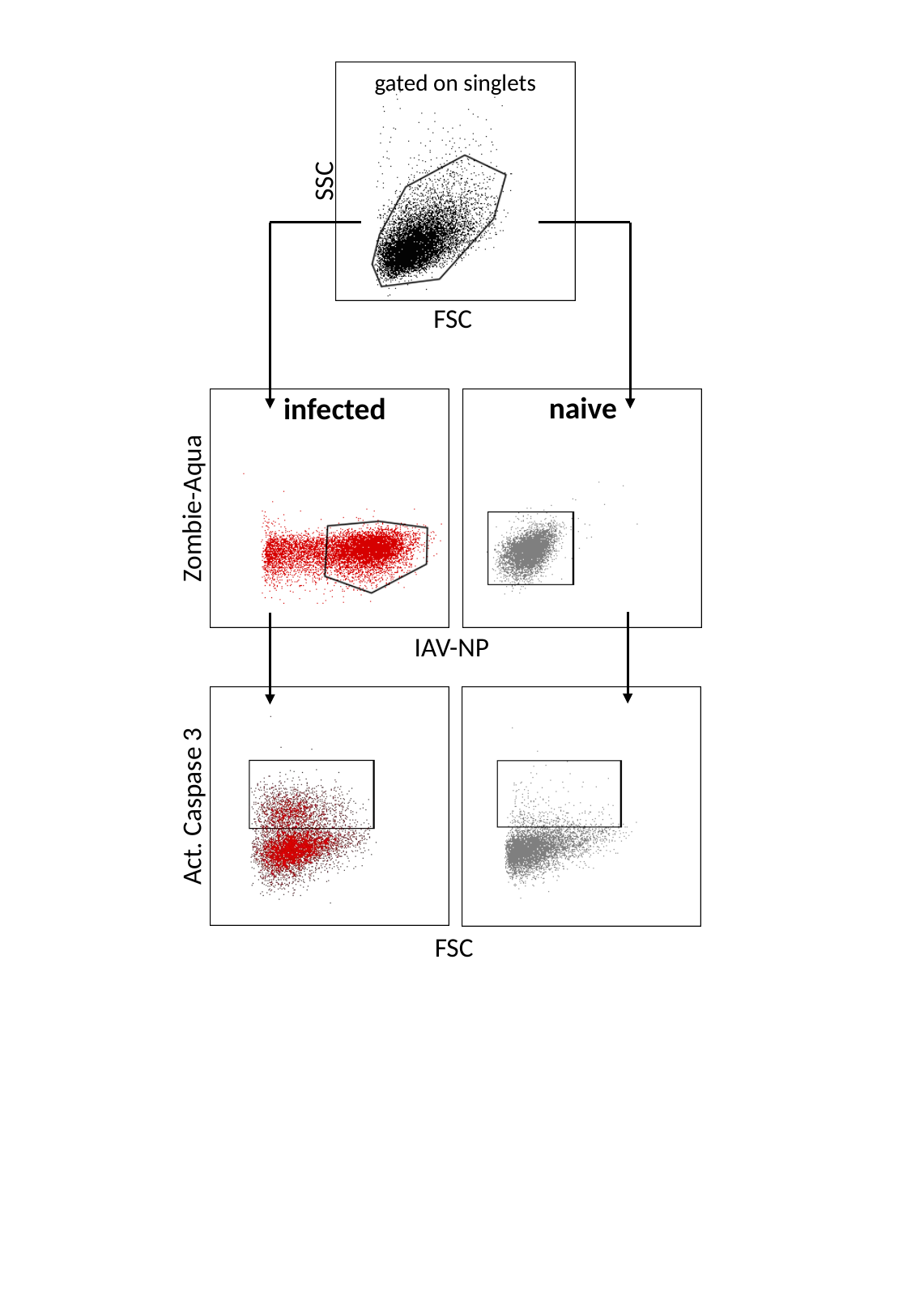

gated on singlets
SSC
FSC
naive
infected
Zombie-Aqua
IAV-NP
Act. Caspase 3
FSC

Supplement: Supplementary file 2 — Additional file 2: Representative Gating strategy for in vitro flow cytometrical phenotyping. Analyses were performed on pre-gated singlets. After selecting the relevant cell population (SSC/FSC) Zombie-negative NP + (infected) or Zombie-negative NP-negative (naïve control) were tested for their aCas3 expression. [file 13567_2023_1257_MOESM2_ESM.pptx]
