# Supplementary material for: The role of PB1-F2 in adaptation of high pathogenicity avian influenza virus H7N7 in chickens
Source: Vet Res. 2024 Jan 3;55:5. doi: 10.1186/s13567-023-01257-8 (PMC10765749; doi:10.1186/s13567-023-01257-8)
Supplement: Supplementary file 3 — Additional file 3: Protein sequence of AR1385 PB1-F2. Highlighted in red is the insertion sight for the introduced mutation in ΔF2. The positions were cytotoxic motifs were previously published are marked grey, the motifs published by Alymova et al. [42] are supplied beneath. For detailed information fasta files are provided (Additional files 4 and 5). [file 13567_2023_1257_MOESM3_ESM.pptx]

## Slide 1
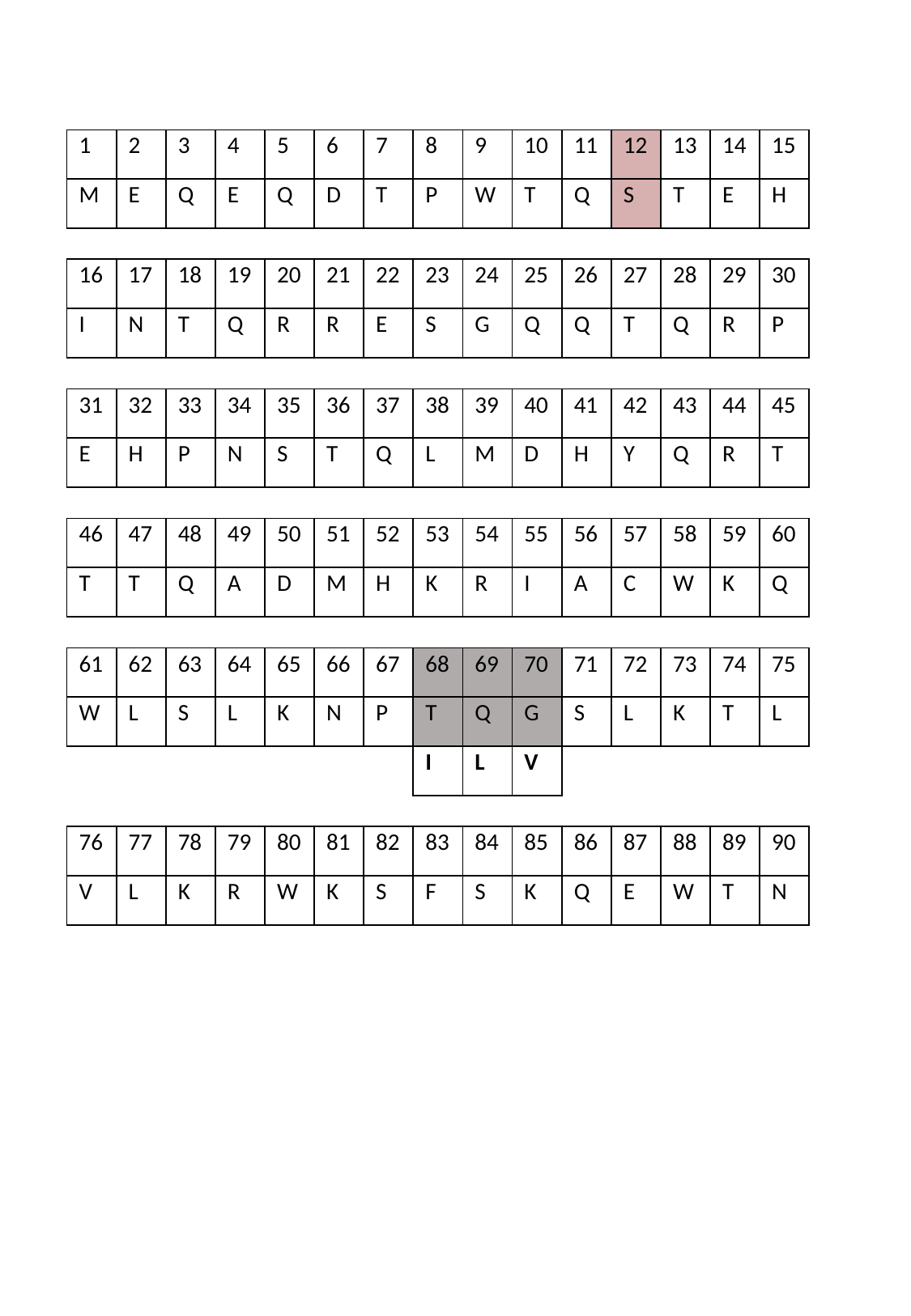

| 1 | 2 | 3 | 4 | 5 | 6 | 7 | 8 | 9 | 10 | 11 | 12 | 13 | 14 | 15 |
| --- | --- | --- | --- | --- | --- | --- | --- | --- | --- | --- | --- | --- | --- | --- |
| M | E | Q | E | Q | D | T | P | W | T | Q | S | T | E | H |
| 16 | 17 | 18 | 19 | 20 | 21 | 22 | 23 | 24 | 25 | 26 | 27 | 28 | 29 | 30 |
| --- | --- | --- | --- | --- | --- | --- | --- | --- | --- | --- | --- | --- | --- | --- |
| I | N | T | Q | R | R | E | S | G | Q | Q | T | Q | R | P |
| 31 | 32 | 33 | 34 | 35 | 36 | 37 | 38 | 39 | 40 | 41 | 42 | 43 | 44 | 45 |
| --- | --- | --- | --- | --- | --- | --- | --- | --- | --- | --- | --- | --- | --- | --- |
| E | H | P | N | S | T | Q | L | M | D | H | Y | Q | R | T |
| 46 | 47 | 48 | 49 | 50 | 51 | 52 | 53 | 54 | 55 | 56 | 57 | 58 | 59 | 60 |
| --- | --- | --- | --- | --- | --- | --- | --- | --- | --- | --- | --- | --- | --- | --- |
| T | T | Q | A | D | M | H | K | R | I | A | C | W | K | Q |
| 61 | 62 | 63 | 64 | 65 | 66 | 67 | 68 | 69 | 70 | 71 | 72 | 73 | 74 | 75 |
| --- | --- | --- | --- | --- | --- | --- | --- | --- | --- | --- | --- | --- | --- | --- |
| W | L | S | L | K | N | P | T | Q | G | S | L | K | T | L |
| | | | | | | | I | L | V | | | | | |
| 76 | 77 | 78 | 79 | 80 | 81 | 82 | 83 | 84 | 85 | 86 | 87 | 88 | 89 | 90 |
| --- | --- | --- | --- | --- | --- | --- | --- | --- | --- | --- | --- | --- | --- | --- |
| V | L | K | R | W | K | S | F | S | K | Q | E | W | T | N |
